# Understanding preferences for self-sampling in a national cervical screening programme: a protocol for a discrete choice experiment

**DOI:** 10.1136/bmjopen-2025-101800

**Published:** 2025-11-27

**Authors:** Shabnam Thapa, Jennifer C Davies, Emma J Crosbie, Katherine Payne, Stuart Wright

**Affiliations:** 1 National Institute for Health and Care Excellence, London, UK; 2 The University of Manchester, Manchester, UK; 3 Division of Cancer Sciences, The University of Manchester, Manchester, England, UK; 4 Department of Obstetrics and Gynaecology, Manchester University NHS Foundation Trust, Manchester, UK; 5 Gynaecological Oncology, Manchester University NHS Foundation Trust, Manchester, England, UK; 6 Manchester Centre for Health Economics, The University of Manchester, Manchester, England, UK

**Keywords:** Patient Preference, Uterine Cervical Neoplasms, Mass Screening

## Abstract

**Abstract:**

**Introduction:**

The National Health Service Cervical Screening Programme (NHSCSP) currently involves a healthcare professional collecting a cervical sample in a healthcare setting. This method of screening has barriers associated with access to screening appointments and the poor acceptability of the speculum examination. Primary screening through HPV testing has led to the development of self-sampling screening methods including vaginal and urine self-sampling, with many UK studies comparing these screening methods with the current NHSCSP. It is not known what features of self-sampling influence individuals’ preferences and cervical screening uptake. To understand these preferences, we plan to undertake a discrete choice experiment (DCE). This protocol aims to describe the steps taken to design the DCE and the proposed approach to fielding the DCE to identify preferences for different sampling approaches in cervical screening.

**Methods and analysis:**

An online survey comprising a DCE was designed to understand preferences of individuals for self-sampling methods within the NHSCSP. Attributes and levels for the DCE were generated through an iterative process including a literature review of qualitative studies about self-sampling cervical screening methods, input from cervical screening clinical experts and a patient and public involvement group (n=6). A D-efficient design was used to create choice sets for the DCE survey. Regression-based analysis will be used to estimate the impact of each attribute and level on individual choices.

**Ethics and dissemination:**

This study has been approved by The University of Manchester Proportionate Research Ethics Committee (2024-20767-37669). The results of the DCE will be submitted for publication in a relevant peer review journal and the results will be presented at national and international conferences.

**Data statement:**

There are no data associated with this protocol. The data produced by this study and analysis scripts will be made available in a public repository following publication of the study.

**STRENGTHS AND LIMITATIONS OF THIS STUDY**

- The attributes and levels will be selected based on previous qualitative work as well as from multiple discussions with relevant stakeholders, including clinicians and public contributors.
- Online recruitment will ensure a broad range of women, who are representative of the demographic composition of the UK, will be able to complete the survey.
- Since the survey will only be available online, there is a potential to miss individuals without technological access and literacy.
- In this study, the survey will only be available in English, limiting access for those who cannot read the language. Further studies are planned to translate the study and field it in groups who are poorly served by current screening programmes.

## Introduction 

 Cervical cancer is one of the most common cancers affecting women and people with a cervix worldwide, with approximately 660 000 new cases annually.^1^ In the UK, around 3200 women are diagnosed with cervical cancer each year, with the highest incidence observed among individuals aged 30–34 years.^2^ Cervical cancer is caused by persistent infection with high-risk human papillomavirus (HPV).^3 4^ HPV is a common virus which is responsible for around 99% of cervical cancer cases.^5^

The current national cervical screening programme (CSP) in the UK targets people with a cervix aged between 25 and 64 years, with a total of 3.43 million attending in 2022–2023.^6^ Those registered with a general practitioner (GP) are invited for screening every 3 or 5 years, depending on age and location within the nations of the UK. At present, screening involves a healthcare professional performing a speculum examination to obtain a cervical sample in a healthcare setting. Since 2019, cervical screening uses primary HPV testing, where the cervical sample is first examined for the presence of the HPV virus. If the sample is HPV negative, no further tests are required and the individual returns to routine recall and will be offered screening in 3 or 5 years. If the result is HPV positive, cytology is performed to triage the individual for further investigations. Depending on the cytology results, the individual may be invited for another screen in a year or referred for a colposcopy if abnormal cells are detected.^7^

Since the introduction of an organised cervical screening programme in England in 1988, cervical cancer deaths have reduced by approximately 70%.^8^ Despite this, the uptake of cervical screening is declining, with only 68.7% of eligible individuals screened in 2022/2023, a decrease of 1.2% from the previous year.^7^

Several barriers to attendance for cervical screening have been identified, including poor access to screening appointments, lack of time to attend screening, poor acceptability of the speculum examination, fear of cancer, low perceived risk and discomfort with the gender of the healthcare professional performing the sampling.^9 10^ Uptake is particularly low among individuals from ethnically diverse backgrounds, those younger than 30 years old, people from socio-economically deprived communities and those who identify as LGBTQ+.^11 12^

To overcome these barriers and increase uptake, there has been growing interest in alternative cervical screening sampling methods such as a self-collected vaginal swab and urine samples. Several countries, including the Netherlands, Malaysia and Albania, offer home-based self-sampling as a primary screening option.^13^,^15^ Other countries such as Australia, Denmark, Finland and France, offer self-sampling to under-screened populations.^16^ UK-based studies are currently being conducted to compare the test performance of self-sampling with healthcare practitioner taken cervical sampling.^17^,^19^ In clinical trials, such as the UK-based HPValidate trial, vaginal self-sampling has been shown to have similar diagnostic accuracy to cervical sampling.^20 21^ However, recent real-world prospective data from the Netherlands, where vaginal self-sampling has been a whole population opt-in option since 2017, shows a decrease in HPV positivity and cervical intraepithelial neoplasia (CIN) 3+ rates in self-sampling compared with healthcare practitioner-taken cervical sampling, which has raised some concern around its comparative test accuracy.^22 23^ Urine self-sampling also shows promising diagnostic test accuracy compared with healthcare practitioner taken cervical sampling if collected with a specialised first void urine collection device.^24 25^ Its high acceptability rate^26^ may potentially improve cervical screening uptake further.

The full potential of cervical screening will only be achieved if there is sufficient uptake. While self-sampling is likely to increase uptake by overcoming some of the barriers of access to cervical screening, the improved uptake would need to be sufficient to offset any decrease in test sensitivity.^27^ Uptake of any service depends significantly on individual preference. It is yet to be understood what characteristics of sampling drive individual choice to attend screening or not. To explore individual preference for self-sampling methods for cervical screening, a discrete choice experiment (DCE) will be conducted.

A DCE is a preference elicitation method used to generate preferences for characteristics (attributes) of an intervention by observing choices made to structured questions.^28 29^ In a DCE, individuals are presented with a series of hypothetical scenarios and asked to choose between different options. These hypothetical scenarios are described by key features of the intervention, known as attributes, and each attribute can have a range of values called levels. The responses to the DCE questions are then analysed to determine preferences, how attributes trade off against each other, and the relative importance of each attribute.^30^ The results from the DCE can be used to predict the potential uptake for different sampling approaches for cervical screening. Heterogeneity in preferences can also be explored, informing the design of a future CSP delivery model that meets the need of the whole population and maximises its uptake and overall impact.

This paper aims to describe the steps taken to design a DCE to understand the preferences of people with a cervix for different attributes of a self-sampling approach within a CSP.

## Methods and analysis

### Overview of approach and methods

An online survey containing a DCE will be designed to elicit preferences from individuals eligible for the CSP. The survey will be developed using guidance for designing preference studies and subsequently reported in line with recently published reporting guidelines.^31 32^ The study will begin on 1 June 2025 and is expected to end by 31 December 2025.

In each DCE task, the participant will be shown two unlabelled alternatives describing potential ways in which a sample could be taken for cervical screening. They will be asked to choose which of the alternatives they would prefer if they had to choose or whether they instead would choose to receive no screening. Participants will be asked to complete a series of these questions and the resulting preference data for all participants will be analysed using econometric models using logistic regression. The results will show which attributes have the biggest impact on driving the decision about whether to have screening and will allow the uptake for specific HPV sampling methods to be predicted.

### Defining the list of attributes and levels

DCEs are underpinned by the theory that when an individual chooses between the available alternatives, they do this based on the attributes of the good or service on offer.^33^ The alternatives represent different hypothetical services that can be chosen by the participant. In this study, the alternatives will be different potential ways of taking the sample for cervical screening. These will be unlabelled, which means they will be described to participants as sampling method 1, sampling method 2 and so on. The alternative would be to use a labelled approach where the alternatives would be labelled as specific sampling approaches, for example, healthcare-practitioner obtained cervical sampling, vaginal self-sampling or urine self-sampling. Our approach was chosen because using a labelled approach would make it impossible to determine the value of some attributes that are directly related to the sampling approach. For example, healthcare practitioner-obtained cervical sampling can never be done in a home setting, so it would not be possible to separate the value of self-sampling as an intervention from the value of being able to collect the sample at home.

Identifying the key attributes and levels of a service is critical to understanding how individuals choose between alternatives. The final list of attributes included in this DCE survey was developed through an iterative process, incorporating information from both published literature and relevant stakeholder input, which follows recommended practice when designing a DCE.^34^

Initially, a list of potential attributes was identified by reviewing qualitative studies on the facilitators, barriers, acceptability, attitudes and perceptions related to various methods of self-sampling methods within a CSP. A systematic approach to searching the literature was conducted in MEDLINE and Embase (up to January 2024) to identify relevant studies. The full search strategy is available in the online supplemental appendix 1. Google Scholar was used to identify any other relevant studies not found in primary databases. This review focused on studies conducted in the UK due to their direct relevance to the decision problem. Four UK-based studies on self-sampling were identified.^935^,^37^ Given the limited number of qualitative studies specific to self-sampling methods in the UK, we expanded our search to include studies from other countries with similar socioeconomic backgrounds (high-income countries as defined by the World Bank) with universal health coverage, ensuring that screening methods are covered by national health systems without additional cost to the users. Exclusion criteria included studies that were not qualitative, did not involve individuals eligible for cervical screening, focused on health provider supply of self-sampling, were unpublished or not available in English. A total of 17 relevant studies were identified, generating a list of 39 potential attributes (see online supplemental appendix 2, table 2.1).

The first step in refining these attributes was to screen them to ensure only those relevant to the decision problem were included. For this initial screening, we used a behavioural screening model, the Integrated Screening Action Model (I-SAM model), as a framework to categorise the attributes according to the seven stages of the screening process: unaware, unengaged, undecided, decided to act, acting, repeat and decided not to act.^38^ The I-SAM model was used as a framework for attribute selection as the decision to undergo screening involves a complex series of stages with an extensive range of influencing factors.

**Figure 1:**
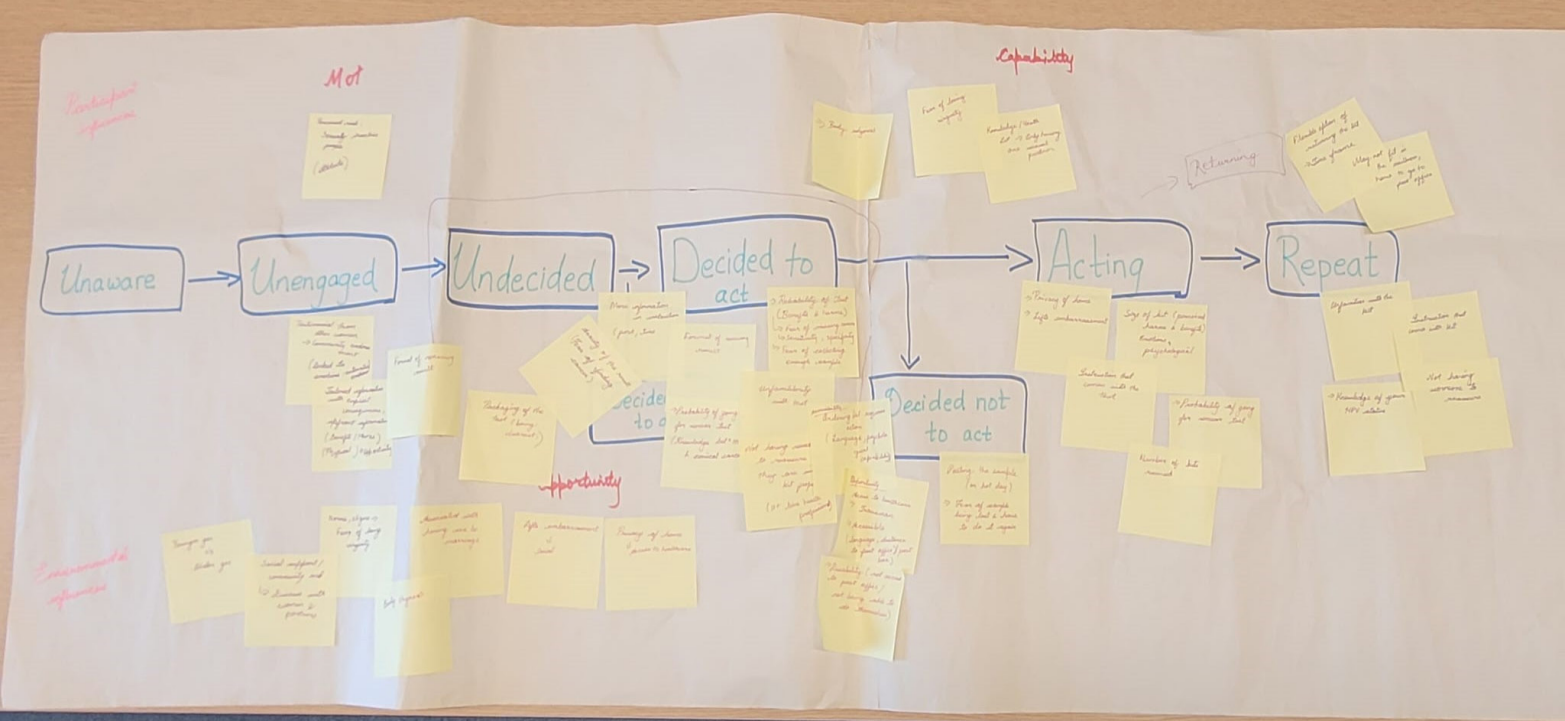
Using the I-SAM framework to assist in attribute selection. I-SAM, Integrated Screening Action Model.

To use the I-SAM to help in attribute selection, two researchers (ST and SW) drew the screening decision pathway on a large sheet of paper (see figure 1 and online supplemental appendix 3). The potential factors influencing cervical screening decision-making identified using the systematic review were written on individual sticky notes. The researchers then took one sticky note at a time and after discussion decided which part of the screening behaviour process pathway that factor would affect. Alternatively, the potential decision factor could be labelled as a participant influence (motivation or capability) or environmental influence (social or physical).

Using the I-SAM model helped to distinguish attributes into those that may be used to make decisions at different stages and factors that might reflect participants’ attitudes, which influence their screening decisions. For example, perception of self-sampling methods might be shaped by education or cultural norms, while motivational factors, such as an understanding of HPV and cervical cancer, can encourage screening. Conversely, physical disabilities might discourage the use of self-sampling methods. While these factors are important, they do not necessarily function as characteristics of the cervical screening methods themselves.

As a result of screening using the I-SAM model, a list of 14 potential attributes was kept. These included: sensitivity of the test; chance of recall; location of the test; method of the test; feeling of discomfort or pain; time taken to receive the results; how to organise an appointment; packaging of the kit; size of the brush; instructions provided with the kit; how the sample is returned; extended time frame of receiving final results for self-sampling; location of self-sampling.

This list of attributes would be too cognitively burdensome to include in a single DCE choice task,^39 40^ with the ideal number of attributes being around six.^41^ However, having too few attributes might also result in excluding a potentially important aspect of the alternative screening methods. The list of attributes was further refined only to include the most important attributes in the DCE survey. To facilitate this refinement, we sought input from relevant stakeholders to reduce the list of attributes and to identify any additional attributes that may have been overlooked. Our stakeholders included: (1) clinical experts with expertise in cervical screening including self-sampling methods, and (2) a patient and public involvement (PPI) group comprising six individuals aged 25–64 years who had previously been invited for cervical screening, representing a diverse range of ethnic backgrounds and including individuals from other groups who face additional barriers in accessing screening, including those with physical disabilities and neurodivergence.

When the initial list of attributes was presented to clinical experts, they did not deem ‘chance of recall’ and ‘time to result’ to be factors that would be used to make a decision about screening method. Hence, these attributes were removed. They also provided feedback on the wording used to describe the attributes. In the PPI group meeting, members were asked their opinion on self-sampling methods and what factors might be important while making choices for cervical screening sampling type. From the PPI group meeting, a new attribute, ‘frequency of screening’ was suggested and subsequently added. This was because one member suggested they may be willing to have more frequent self-sampling if it could offset the reduced test sensitivity. The levels of the attributes were set with input from the clinical experts. The likelihood of detecting cervical abnormalities was set to cover the range of potential sensitivities for different types of self-sampling.^22^ The potential intervals for sampling were aligned to different intervals used in the National Health Service (NHS) Cervical Screening Programme for HPV positive individuals (1 year) or HPV negative individuals (3 or 5 years). 2-year and 1-year intervals were added to represent the potential to shorten intervals to offset the reduced sensitivity of self-sampling. The final list of levels and attributes is presented in table 1.

**Table 1:** Final list of attributes and levels

Attributes	Description	Levels	Attribute coding
Likelihood the sampling method detects cervical abnormalities if present	How accurate is the test to detect abnormalities in cervical cells?	80% 85% 90% 95%	Continuous
How is the sample taken?	Process of taking the sample	Healthcare practitioner takes the sample from the cervix using a speculum Healthcare practitioner takes a vaginal swab sample Self-taken vaginal swab sample Urine sample	Categorical
Location to take the sample	Place where the sample will be taken	Home Healthcare setting	Categorical
Feeling of discomfort and/or pain	Feeling of discomfort or pain while taking the sample	No discomfort or pain Mild discomfort and/or pain Moderate discomfort and/or pain Severe discomfort and/or pain	Categorical
Contact with healthcare practitioner	Opportunity to talk with healthcare practitioner	No contact Contact via phone Contact in person	Categorical
Frequency of screening	How often will the sample be taken for screening?	Yearly Every 2 years Every 3 years Every 5 years	Continuous

In the time since the attribute selection process was conducted, a further study exploring women’s views of self-sampling for non-attenders has been published in the UK.^42^ This study involved women completing a postal questionnaire delivered alongside a self-testing kit as part of a clinical trial. As part of this questionnaire, free-text comments were collated and analysed to identify key themes in responses. These included the ease of use of the test, the positive experience of self-sampling, issues with self-sampling, worries about test accuracy and self-confidence in test completion. The themes identified had been covered in the other studies identified in the review in this paper, so no additional attributes were considered in the DCE design.

### Creating an experimental design

Experimental design is the process through which distinct combinations of attributes and their corresponding levels are transformed into choice sets in which participants express their preferences by making choices.^43^ An effective experimental design ensures that preferences for all of the attributes and levels can be estimated while minimising uncertainty in the results. When all possible combinations of attribute levels are included in the choice sets, this is known as full factorial design. Taking the attributes and levels presented in table 1, the full factorial for the choice task would give 1152 potential questions and 1 327 104 choice sets which is excessively large for practical use and might also allow occurrence of illogical combinations.

To reduce the number of choice sets to a manageable size to prevent placing excessive cognitive demands on the participants, D-efficient designs were generated using Ngene software.^44^ The D-efficient design was designed with restrictions to prevent illogical combinations of attributes and levels from occurring together. For example, a healthcare practitioner would not be able to take the sample in a patient’s home, so these levels were prevented from occurring together.

A total of 24 questions were designed and divided into three blocks with 8 questions per block. The survey was designed to identify main effects only. At the end of the choice task questions, four DCE tasks were included with fixed attributes representing different ways that cervical screening could be offered in practice. These included tasks with a realistic representation of healthcare-practitioner obtained cervical sampling versus no screening; a realistic representation of vaginal self-sampling versus no screening; and a choice of realistic representations of healthcare practitioner-obtained cervical sampling or vaginal self-sampling; and finally a choice of a realistic representation of healthcare-practitioner sampling, vaginal self-sampling and urine self-sampling. The aim of these questions is to determine whether uptake for screening is likely to be higher if a choice of sampling method is offered compared with a recommendation that everyone receives the same type of screen.

### Designing the survey

The survey questionnaire will include five sections. The first section will include a participant information sheet explaining the study and will include a question that asks participants if they consent to taking part in the study. The next section will introduce the study, providing background information about the purpose of the study. The second section will offer detailed information on cervical cancer and different methods of collecting samples for cervical screening. This information will be designed using current NHS information leaflets for cervical screening and adapted to include information about self-sampling methods. The information will be checked for completeness and readability by members of a public advisory group (n=2). This information is intended to equip participants with the necessary knowledge to make informed decisions in the section following.

The third section will be the DCE questions where each participant will be asked to answer a series of choice questions. This will include eight choice questions with varying attributes and levels and then four supplementary questions showing fixed scenarios. First, each attribute and corresponding levels will be described in detail and then the choice questions will be presented to the participants. The final section will gather additional socio-demographic information including: age; ethnicity; religion; employment status; education; disability; sexual orientation; gender; whether they have been sexually active in the past; whether they have been invited to cervical screening in the last 5 years; whether they have attended cervical screening in the last 5 years; awareness that screening uses HPV testing; views about self-sampling; sources of health information; and degree of risk-seeking behaviour. The full survey will be designed in the survey software Qualtrics and fielded online.

**Figure 2:**
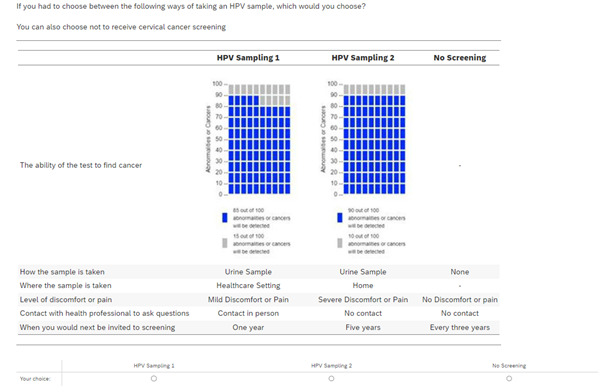
Example choice question. HPV, human papillomavirus.

An example of a choice question that will be presented in the DCE section of the survey is presented in figure 2.

### Piloting of the survey

Before fielding a DCE in a full sample, piloting is required to test the survey. Piloting can improve the survey by checking participant understanding of the description of attributes and levels, length of survey, adequacy of design and data collection ensuring that the data can be optimally analysed.

To see if there is any adjustment needed to the final survey draft, a pre-pilot study will be conducted using face-to-face think-aloud interviews with members of the public advisory group. During these interviews, participants will be asked to share their thought process while completing the survey. This approach allows researchers to understand the aspects of the survey where participants might misunderstand the questions or find them unclear.^45^ By applying the think-aloud approach, researchers can identify potential issues and make necessary adjustments to improve the survey. Following the pre-pilot, a quantitative pilot study will be conducted with a sample of 50 participants, including individuals from ethnically diverse backgrounds (such as Asian, black, Caribbean and Arab). This pilot will check for design and coding errors, ensuring that all model coefficients can be estimated.

### Participant sampling and recruitment

There is no set rule on how many participants can be recruited for the DCE as estimating sample size for a DCE is quite challenging (17). The sample size depends on several factors such as the number of attributes and levels, choice tasks, continuous and categorical variables and the degree of heterogeneity in responses. A sample of around 1000 is aimed for this study to increase the power of the study as well as ensure sufficient power for investigating heterogeneity of preferences in different groups.

The relevant study population will be individuals who have a cervix and are between the ages of 25–64 years, and therefore eligible for cervical screening. The target population will be recruited by a commercial participant panel recruiter who will send a survey link to potential participants.

### Quality control

It is necessary to ensure that the responses received from participants represent their true preferences as closely as possible. It is possible for DCE surveys to receive fraudulent responses, particularly when fielded online. Also, respondents may complete the survey quickly without fully reading all of the information or only focus on certain attributes and levels. Such responses, when included in the analysis, can result in biased results. Although econometric models account for random respondents, retaining these inaccurate responses can potentially create concern for the results of other survey questions.

To mitigate this risk, several quality control measures will be taken using tools in-built in Qualtrics: responders completing the survey too quickly (more than two SDs from the median) will be flagged, invisible Captcha technology will be used to help flag responses from bots, and a flag will be added to responses exhibiting potential straightlining (choosing the same option for all DCE tasks). Data analysis will be conducted with flagged responses included, with additional sensitivity analysis conducted with flagged responses excluded.

### Analysis approach

Data analysis will be conducted in R software using the Apollo package.^46^ The response rate will be calculated using data on the number of survey invitations distributed by the panel provider, the number of participants who opened the survey and the number of participants who completed the survey. Participants’ demographic information and responses to attitudinal questions will be summarised. As demographic questions are included at the end of the survey, and no personal information is sent by the panel provider to the research team, it will not be possible to analyse whether certain demographic groups were more likely than others to drop out of the survey. The number of participants flagged as potentially fraudulent will be reported. The results of the DCE questions with fixed attributes for different realistic screening scenarios will be reported indicating the proportion of respondents who would choose healthcare professional sampling compared with no sampling, self-sampling compared with no sampling, healthcare professional or self-sampling compared with no sampling and vaginal self-sampling compared with urine self-sampling and healthcare professional self-sampling.

For each DCE choice task, a process will be undertaken to select the functional form and model approach that best explains the data. A conditional logistic regression model will be estimated for each DCE with categorical variable effects coded, continuous variables assumed to be linear and a single constant included to represent the probability of opting in versus opting out. Different model functional forms will be estimated whereby two constants are used to represent the probability of selecting hypothetical risk prediction or feedback scenario A or scenario B. This serves as a test as to whether participants were always choosing scenario A or B regardless of the levels shown. All tests of model specification will be made by comparing the Bayesian information criterion (BIC) of the different models. If a model specification is found to result in a lower BIC value then this suggests that the model specification adds sufficient additional explanatory power for the number of additional parameters in the model. 

Analytical tests will be conducted to determine if there is evidence of non-linearity in the continuous variables (see table 1) and y in the model. Each continuous variable will be coded in quadratic, log and piece-wise forms to determine if any of these specifications reduces the BIC. On selection of the final functional form for the model, random parameter logit models will be estimated to determine if a model which allows for preference heterogeneity provides a better fit for the data. First, an uncorrelated random parameter logit will be estimated for each DCE, and a fully correlated random parameter logit will be estimated. The fully correlated model allows for differences in error between participants as well as differences in preferences. 

To investigate heterogeneity in preferences for sampling approach, latent class analysis will be used to identify groups of participants with similar preferences. The number of classes to include will be decided using an iterative approach comparing the BIC of each model as classes are added until the BIC no longer reduces. A further model will be fit to determine if any demographic variables are correlated with class membership.

Using the best model as determined using the BIC, the uptake for different potential sampling approaches for cervical screening will be estimated. The potential uptake for different approaches to sampling in the latent class groups will also be estimated.

### Limitations

There are some limitations to this study arising from the use of an online panel of women. While a sample can be recruited that is theoretically representative of members of the UK in terms of key demographic factors such as education and ethnicity, the use of an online survey means that individuals without access to the internet or a device to complete the survey are excluded. In addition, in this study, the survey will only be available in English, limiting access for those who do not read English. Both of these factors are important limitations as the groups who may face barriers to accessing the survey are also those who are likely to face barriers to accessing screening.

A second stage of the research project will seek to address these barriers by explicitly adapting and fielding the survey in groups who are currently poorly served by cervical screening. This currently includes plans to field the survey in the Bangladeshi community and among members of the LGBTQIA+community. Details of how these groups will be approached have not been included in this study as the methods for adapting and fielding the survey will be significantly different to this larger population study. For example, for fielding the survey in the Bangladeshi community, the survey will be translated into Bengali (with support from a Bengali speaking PPI member) and fielded in person by a community researcher using paper surveys. The development of this approach is being supported by a community organisation supporting the Bangladeshi community.

### Patient and public involvement

A public and patient involvement group comprising six women with experience of being invited to cervical screening provided input into the design of this study. An online focus group was held with the PPI group to discuss the key attributes which would impact their decision to attend screening using different sampling methods. This helps refine the long list of initial attributes and resulted in an additional attribute (screening intervals) being added. In addition, three members of the PPI group provided input into the design and content of the information materials to be included in the DCE study.

### Ethics and dissemination

Proportionate ethical approval for this study has been granted by The University of Manchester Research Ethics Committee. A participant information sheet will be shown on the first accessed digital page of the study platform and participants will be asked for consent to take part in the study. The participant information sheet will inform participants that the data from this study will be made publicly available on submission of the work to a scientific journal. The survey will not explicitly record any personal information about participants and any free-text comments will be removed before the data is stored in an open access repository.

The results of the DCE will be published in a peer-reviewed journal. A Shiny package created using the software R will be used to make an application which will allow decision-makers, such as the UK National Screening Committee, to combine different attributes and levels and explore the potential uptake for the resulting sampling approaches as predicted by the model. This will include uptake in the different latent class groups.

## Supplementary material

10.1136/bmjopen-2025-101800 online supplemental appendix 1
