# Supplementary material for: Understanding preferences for self-sampling in a national cervical screening programme: a protocol for a discrete choice experiment
Source: BMJ Open. 2025 Nov 27;15(11):e101800. doi: 10.1136/bmjopen-2025-101800 (PMC12666229; doi:10.1136/bmjopen-2025-101800)

**Supplementary Materials**

**Understanding preferences for self-sampling in a national cervical screening programme: a protocol for a discrete choice experiment**

Shabnam Thapa^1,2^, Jennifer C. Davies^3, 4^, Emma J. Crosbie^,3, 4^, Katherine Payne^2^, Stuart J. Wright^2*^

^1^National Institute for Health and Care Excellence, Manchester, UK

^2^Manchester Centre for Health Economics, The University of Manchester, Manchester, UK

^3^Gynaecological Oncology Research Group, Division of Cancer Sciences, Faculty of Biology, Medicine and Health, University of Manchester, Manchester, UK.

^4^Department of Obstetrics and Gynaecology, Manchester Academic Health Science Centre, St Mary's Hospital, Manchester University NHS Foundation Trust, Manchester, UK.

Correspondence to: Dr Stuart Wright. Jean McFarlane Building, The University of Manchester, Oxford Road, Manchester, M13 9PL. stuart.j.wright@manchester.ac.uk

Supplementary appendix 1

Search Strategy

Ovid MEDLINE(R) <1946 to January Week 5 2024>

1 qualitative studies.mp. or Qualitative Research/

2 Focus Groups/

3 Interview/

4 Observation/

5 ethnography.mp.

6 Grounded Theory/

7 acceptability.mp.

8 Health Knowledge, Attitudes, Practice/ or facilitators.mp.

9 barriers.mp.

10 challenge*.mp.

11 preference*.mp.

12 acceptance.mp.

13 facilitate*.mp.

14 self sampl*.mp.

15 self collect*.mp.

16 self administered.mp.

17 self test*.mp.

18 self evaluation.mp. or Diagnostic Self Evaluation/

19 HPV.mp. or Papillomavirus Infections/ or Uterine Cervical Neoplasms/

20 cervical.mp.

21 cervix.mp. or Cervix Uteri/

22 cervical cancer.mp. or Uterine Cervical Neoplasms/

23 1 or 2 or 3 or 4 or 5 or 6 153348

24 7 or 8 or 9 or 10 or 11 or 12 or 13

25 14 or 15 or 16 or 17 or 18

26 19 or 20 or 21 or 22

27 23 and 24 and 25 and 26

Embase <1974 to 2024 Week 06>

1 qualitative studies.mp. or qualitative research/

2 structured interview/ or telephone interview/ or audio interview/ or interview.mp. or unstructured interview/ or video interview/ or semi structured interview/ or interview/

3 participant observation/ or observation/

4 ethnography/ or ethnology/

5 grounded theory/

6 acceptability.mp. or program acceptability/

7 facilitators.mp.

8 barriers.mp.

9 challenge*.mp.

10 preference*.mp. or social preference/ or patient preference/

11 acceptance.mp.

12 facilitate*.mp.

13 sampling/ or self sampl*.mp.

14 self collect*.mp.

15 self administered.mp.

16 self-testing/ or self test.mp.

17 self evaluation/

18 HPV.mp. or Wart virus/

19 Papillomavirus.mp. or Papillomaviridae/

20 uterine cervix cancer/ or cancer screening/

21 uterine cervix/

22 1 or 2 or 3 or 4 or 5

23 6 or 7 or 8 or 9 or 10 or 11 or 12

24 13 or 14 or 15 or 16 or 17

25 18 or 19 or 20 or 21

26 22 and 23 and 24 and 25

**Supplementary materials 2: Attribute identification**

**Supplementary Table 2.1 Long list of attributes derived from literature revie**

| **Cultural aspect** | **Device itself** | **Social** | **Communication** | **Knowledge aspect** | **Trans man/non-binary** | **Others** |
| --- | --- | --- | --- | --- | --- | --- |
| Sexually inactivity (including widows, unmarried women etc) believing that they do not need screening | Reliability of the test | Younger generation versus older generation – older generation reluctant to use | Testimonials from other women in the information sheet | Link between HPV positivity and cervical cancer | Genital dysphoria – would increase by having to engage with own genitalia | Ordering kit requires action - barrier to people with language, disability |
| Fear of losing virginity | Size of the sampling kit – small preferred | Discuss with other women and partners | Tailored information with explicit consequences of late detection in invitation letter | The test is for HPV and cervical sample will be needed if the result is HPV positive | Genital dysphoria – would decrease by not having to expose their genitals to someone else | Anxiety of the result |
| Associated with sexual activity before marriage | Confidence in doing the screening/Fear of not doing it correctly |  | Invitation letter – upfront information on test accuracy |  | Accessibility | Flexible option of returning the kit |
| Unmarried women living with family might have dilemma to have the kit sent to their home address | Instructions that come with sampling method |  | More information in instruction leaflet – how to store the sample and post (eg. posting the sample on a hot day), timeframe to return the sample, reminder to post |  |  | May not fit in the mailbox, have to go to the post office |
| Privacy of home | Unfamiliar with the kit |  | Format of receiving result |  |  | Fear of detecting cancer |
| **Cultural aspect** | **Device itself** | **Social** | **Communication** | **Knowledge aspect** | **Trans man/non-binary** | **Others** |
| Alleviates embarrassment | Not having someone to reassure them that they are using the kit properly |  | Fear of post office losing the sample and having to go through doing the test again |  |  |  |
| Only having one sexual partner mean you can’t get HPV/cervical cancer | Fear of increasing infection – if the device is lost inside the body |  |  |  |  |  |
| Body shyness, unwillingness to touch or engage with own genitals | Risk of hurting oneself if not done correctly |  |  |  |  |  |
|  | Fear of not collecting enough sample |  |  |  |  |  |
|  | Fear of doing the sampling wrong and getting the wrong result |  |  |  |  |  |
|  | Incorrect belief that they may not able to collect sample properly as they might not reach their cervix with the swab |  |  |  |  |  |
|  | Sterilisation of the swab |  |  |  |  |  |
|  | First time maybe shown by health professional |  |  |  |  |  |
|  | Fear of missing cancer |  |  |  |  |  |

Supplementary appendix 3:

Detailed images of the I-SAM model for attribute selection


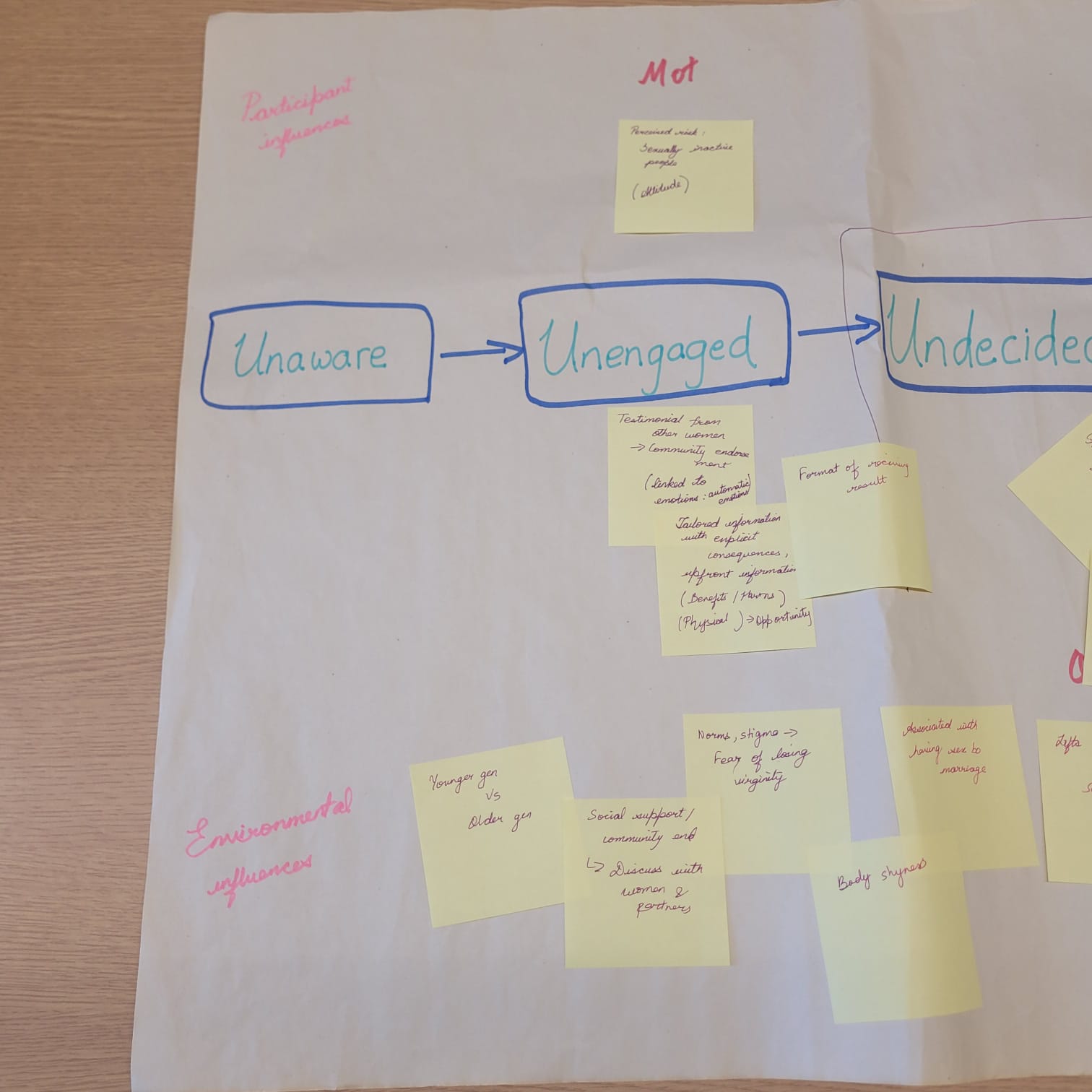

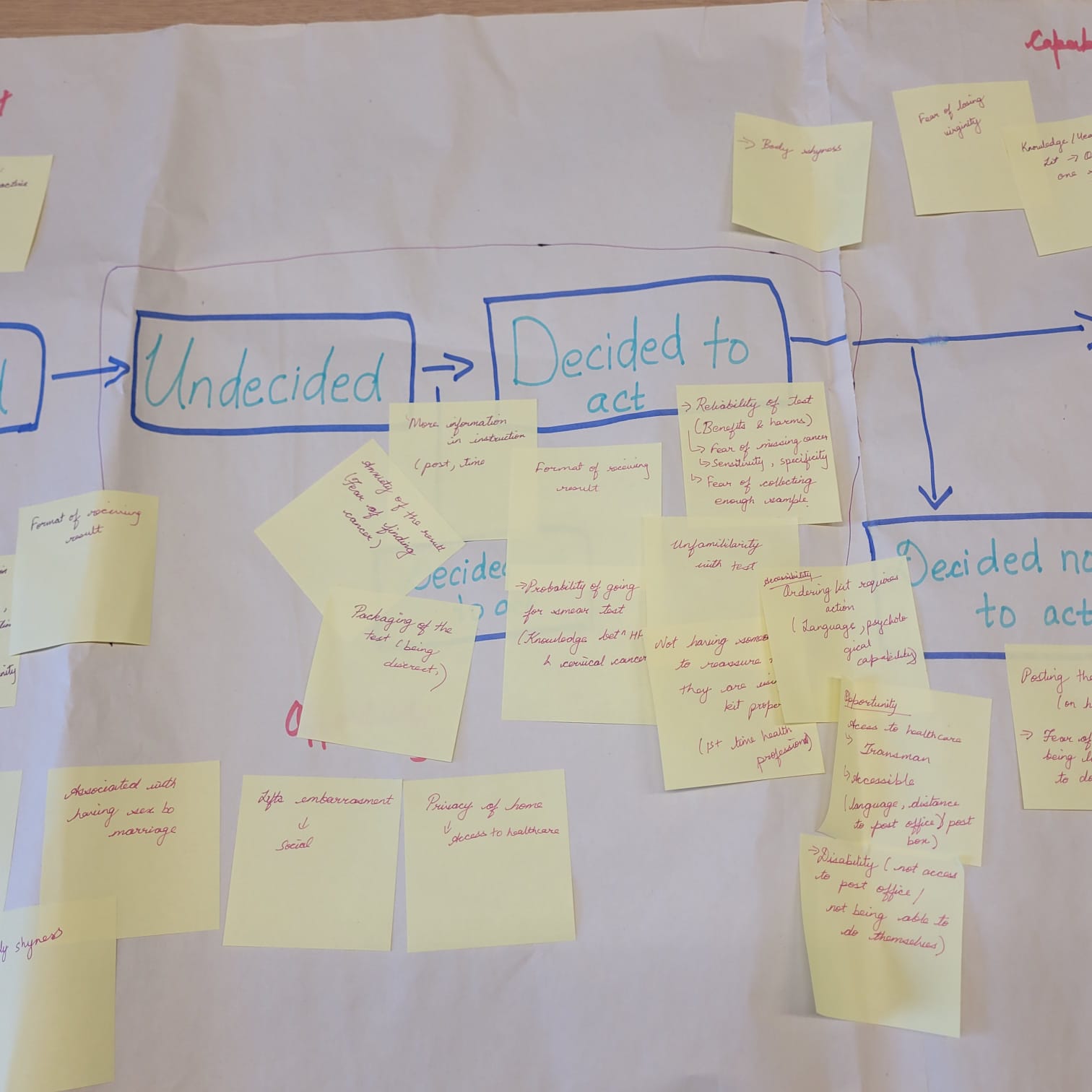

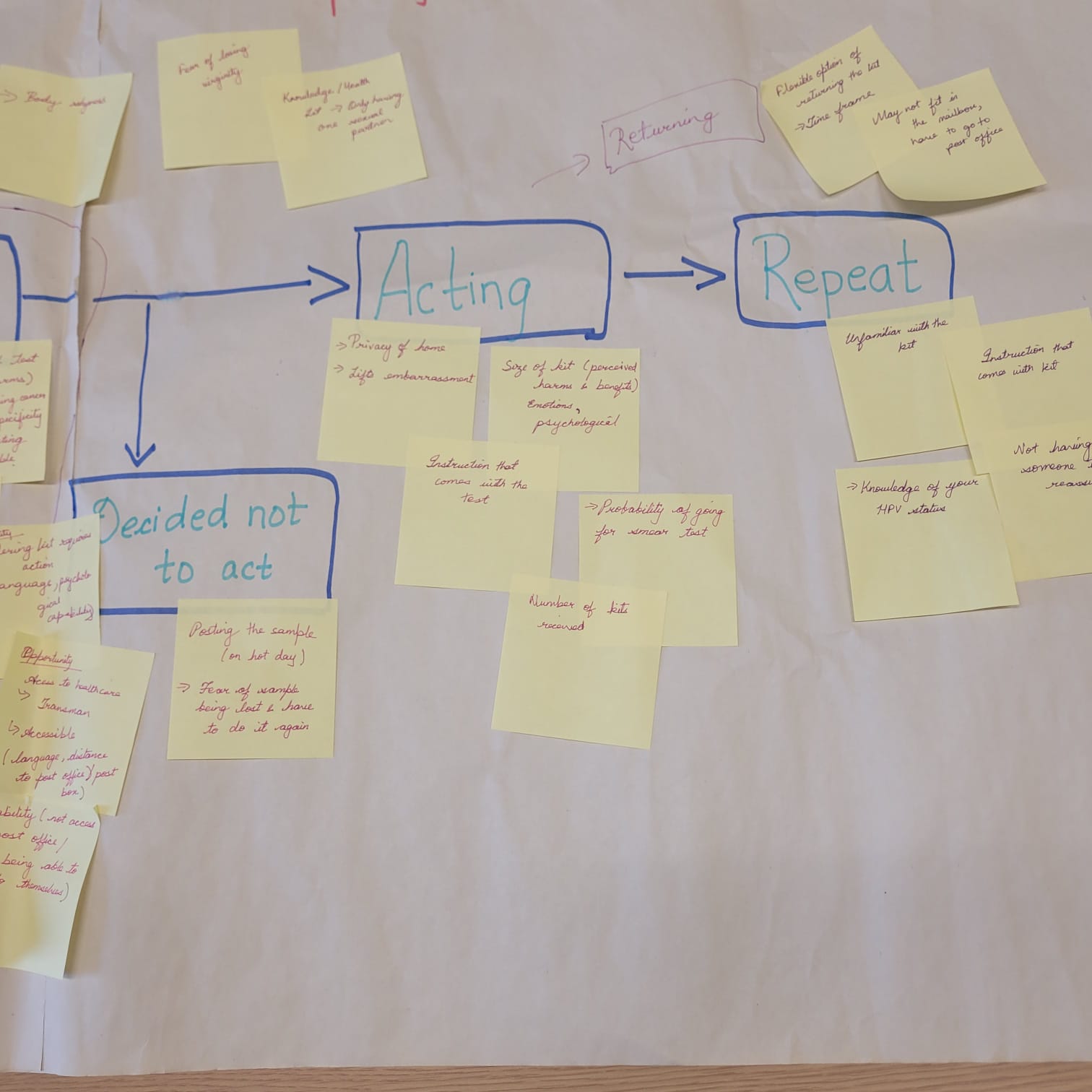

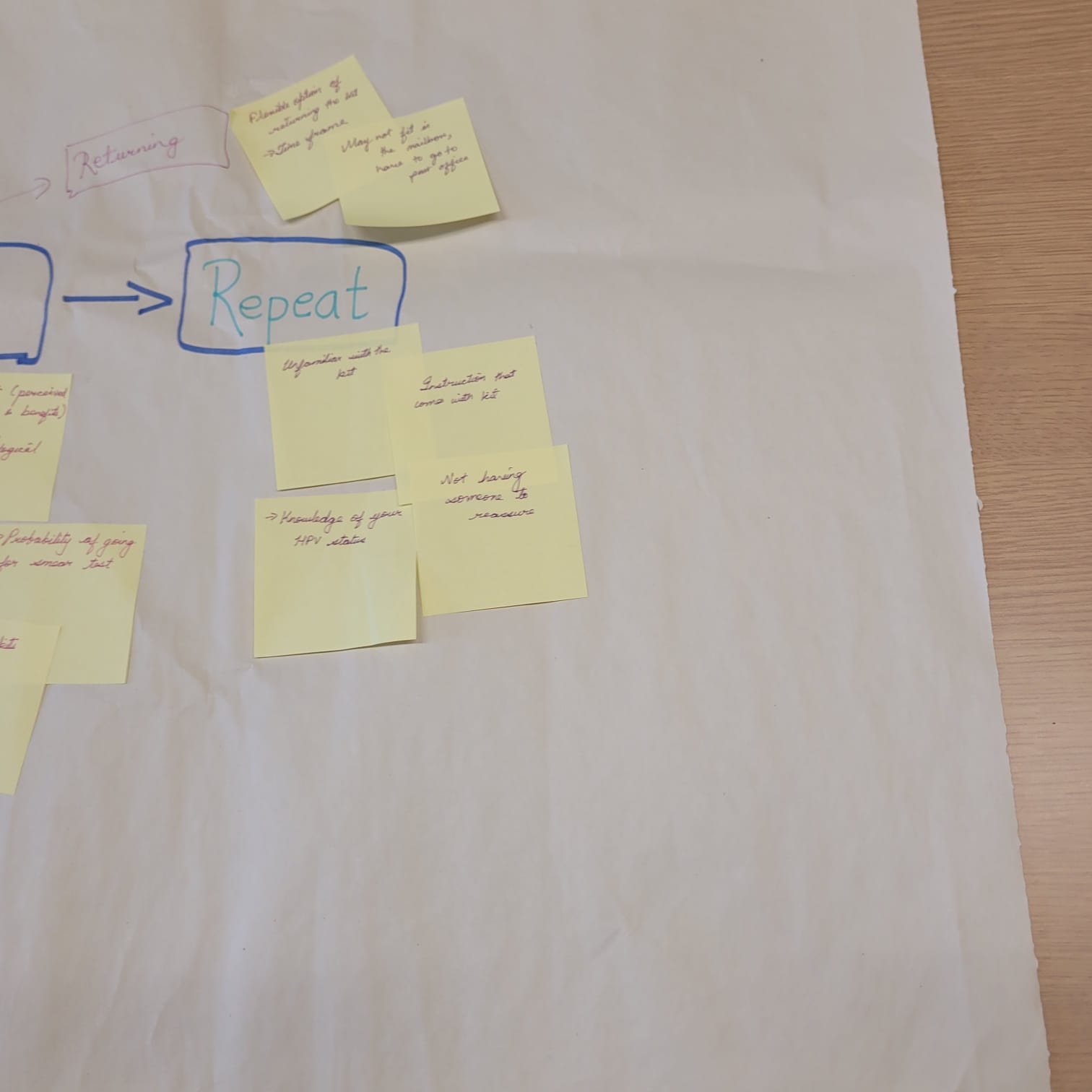

Supplement: online supplemental appendix 1 [file bmjopen-15-11-s001.docx]
