# Supplementary material for: Understanding preferences for self-sampling in a national cervical screening programme: a protocol for a discrete choice experiment
Source: BMJ Open. 2025 Nov 27;15(11):e101800. doi: 10.1136/bmjopen-2025-101800 (PMC12666229; doi:10.1136/bmjopen-2025-101800)
Supplement: Supplementary file 2 [file bmjopen-2025-101800.reviewer_comments.pdf]

PEER REVIEW HISTORY

BMJ Open publishes all reviews undertaken for accepted manuscripts. Reviewers are asked to complete a checklist review form and are provided with free text boxes to elaborate on their assessment. These free text comments are reproduced below.

ARTICLE DETAILS

Title (Provisional)

Understanding preferences for self-sampling in a national cervical screening programme: a protocol for a discrete choice experiment

Authors

Thapa, Shabnam; Davies, Jennifer C.; Crosbie, Emma J; Payne, Katherine; Wright, Stuart

---

VERSION 1 - REVIEW

**Reviewer**	**1**
**Name**	**Berner, Alison**
**Affiliation**	**Tavistock and Portman NHS Foundation Trust**
**Date**	**27-Aug-2025**
**COI**	**I am co-author on two manuscripts with Emma Crosbie and Jennifer Davies which have not yet been reviewed for publication but have had no input in this manuscript or the project it relates to.**

Many thanks for this manuscript which clearly describes the planned study.

I have little to add except that it is (often necessarily due to methods) lengthy in places.

I would therefore suggest that the authors review for repetition in "Creating an Experimental Design" and "Designing the Survey" sections as there is some around how questions will be delivered.

In addition, a diagram on how the methodology and design relates to the I-SAM model may be useful.

**Reviewer**	**2**
**Name**	**Carlander, Christina**
**Affiliation**	**Karolinska University Hospital**
**Date**	**12-Sep-2025**

COI **None**

The authors wish to asses what features of self-sampling that influence

individual's preferences for self-sampling and cervical screening uptake which is important for the update of current screening programs. The study-protocol describes a planned discrete choice experiment (DCE) and the proposed approach to fielding the DCE to identify preferences for different

sampling approaches in cervical screening.

*Abstract-ok, no comments

*Introduction-ok, no comments

*Methods and anlysis

Q1. In the Introduction the authors conclude "Uptake is particularly low among individuals from ethnically diverse backgrounds, those younger than 30 years old, people from socio-economically deprived communities and those who identify as LGBTQ".

I lack a description of how you intend to ensure that those mentioned above who are at particularly high risk of low uptake are included in the study population. If there is no such plan this should be included under Limitations

Q2. Will the survey be available in other languages than English? If not this should be mentioned under Limitations and how this may limit the number of migrants included in the study-a group known to have lower uptake of cervical screening.

VERSION 1 - AUTHOR RESPONSE

Reviewer: 1
Dr. Alison Berner, Tavistock and Portman NHS Foundation Trust
Comments to the Author:
Many thanks for this manuscript which clearly describes the planned study.

I have little to add except that it is (often necessarily due to methods) lengthy in places.

I would therefore suggest that the authors review for repetition in "Creating an Experimental Design" and "Designing the Survey" sections as there is some around how questions will be delivered.

Response: *having re-read these sections we have noted that we described the inclusion of the fixed choice scenarios in both sections with virtually identical text. We have now removed this from the designing the survey section.*

In addition, a diagram on how the methodology and design relates to the I-SAM model may be useful.

***Response**: we have added some additional text to explain how we practically used the I-SAM model to help with attribute selection. We have also added a picture of the physical sheet we used to manually do this as we believe this would be of interest to other researchers who may wish to try this approach.*

To use the I-SAM to help in attribute selection, two researchers (ST and SJW) drew the screening decision pathway on a large sheet of paper (see figure 1). The potential factors influencing cervical screening decision making identified using the systematic review were written on individual sticky notes. The researchers then took one sticky note at a time and after discussion decided which part of the screening behaviour process pathway that factor would affect. Alternatively, the potential decision factor could be labelled as a participant influence (motivation or capability) or environmental influence (social or physical).

Reviewer: 2
Dr. Christina Carlander, Karolinska University Hospital
Comments to the Author:
The authors wish to asses what features of self-sampling that influence individual's preferences for self-sampling and cervical screening uptake which is important for the update of current screening programs. The study-protocol describes a planned discrete choice experiment (DCE) and the proposed approach to fielding the DCE to identify preferences for different
sampling approaches in cervical screening.

*Abstract-ok, no comments

*Introduction-ok, no comments

*Methods and anlysis

Q1. In the Introduction the authors conclude ”Uptake is particularly low among individuals from ethnically diverse backgrounds, those younger than 30 years old, people from socio-economically deprived communities and those who identify as LGBTQ”.
I lack a description of how you intend to ensure that those mentioned above who are at particularly high risk of low uptake are included in the study population. If there is no such plan this should be included under Limitations

Response: *we agree with both of the reviewer's comments and it was an oversight not to include a limitations section in this manuscript. We have now added this section. We have also noted that while this study aims to field the survey in a large representative population of the UK, further studies are planned to adapt the survey for different communities who are poorly served by cervical screening programmes. As the methods*

for these surveys will be significantly different. They are considered as separate studies but we believe it is still important to mention them here:

There are some limitations to this study arising from the use of an online panel of women. While a sample can be recruited that is theoretically representative of members of the UK in terms of key demographic factors such as education and ethnicity, the use of an online survey means that individuals without access to the internet or a device to complete the survey are excluded. In addition, in this study the survey will only be available in English, limiting access for those who do not read English. Both of these factors are important limitations as the groups who may face barriers to accessing the survey are also those who are likely to face barriers to accessing screening.

A second stage of the research project will seek to address these barriers by explicitly adapting and fielding the survey in groups who are currently poorly served by cervical screening. This currently includes plans to field the survey in the Bangladeshi community and among member of the LGBTQIA+ community. Details of how these groups will be approached have not been included in this study as the methods for adapting and fielding the survey will be significantly different to this larger population study. For example, for fielding the survey in the Bangladeshi community, the survey will be translated into Bengali (with support from a Bengali speaking PPI member) and fielded in person by a community researcher using paper surveys. The development of this approach is being supported by a community organisation supporting the Bangladeshi community.

Q2. Will the survey be available in other languages than English? If not this should be mentioned under Limitations and how this may limit the number of migrants included in the study-a group known to have lower uptake of cervical screening.

***Response**: please see response to above comment*

Reviewer: 1
If you have selected ‘Yes’ above, please provide details of any competing interests.: I am co-author on two manuscripts with Emma Crosbie and Jennifer Davies which have not yet been reviewed for publication but have had no input in this manuscript or the project it relates to.

Reviewer: 2
If you have selected ‘Yes’ above, please provide details of any competing interests.: Not applicable

VERSION 2 - REVIEW

**Reviewer**	**1**
**Name**	**Berner, Alison**
**Affiliation**	**Tavistock and Portman NHS Foundation Trust**
**Date**	**11-Oct-2025**

COI

Many thanks for the authors' revisions. I feel this is ready for publication.

**Reviewer**	**2**
**Name**	**Carlander, Christina**
**Affiliation**	**Karolinska University Hospital**
**Date**	**01-Oct-2025**
**COI**	

I am happy with the author response and have no further questions
